# EULAR Rheumatology Open Special Issue titled ‘VSI: ATT2026’: the role of clonal haematopoiesis in immunological and rheumatological diseases

**DOI:** 10.1016/j.ero.2026.100232

**Published:** 2026-08-31

**Authors:** Kenneth Walsh, Ariel H Polizio

**Affiliations:** Division of Cardiovascular Medicine, University of Virginia, Charlottesville, VA, USA

## Abstract

Somatic mutations progressively accumulate in all tissues with age. Although most of these acquired mutations have no consequences on the cell's phenotype, some may occur in genetic regions that provide a small growth, survival, or self-renewal advantage to the mutant cells. These ‘driver’ gene mutations will lead to the clonal expansion of mutant cells. When clonal expansions occur in the blood system, they are referred to as ‘clonal haematopoiesis’ or ‘clonal haematopoiesis of indeterminate potential’ to distinguish these cellular expansions from overt haematological disease. The incidence of detectable clonal haematopoiesis increases with age, with relatively large clones detected using standard next-generation sequencing occurring in >10% of the population by 70 years of age. However, with the advent of error-corrected DNA sequencing, more recent studies have shown that smaller clones can be detected in most individuals by middle age and that this condition is an inevitable consequence of ageing. Emerging evidence reveals that the expansion of these mutant clones drives a systemic inflammatory state, significantly impacting the pathogenesis, severity, and therapeutic response across a spectrum of cardiovascular and autoimmune diseases. Additional clinical and experimental studies are warranted to address outstanding questions regarding disease causality, clinically relevant clone sizes, and driver gene-specific effects in these conditions.

Somatic mutations progressively accumulate in all tissues with age. Although most of these acquired mutations have no consequences on the cell’s phenotype, some may occur in genetic regions that provide a small growth, survival, or self-renewal advantage to the mutant cell. These ‘driver’ gene mutations will lead to the clonal expansion of the mutant cells. When clonal expansions occur in the blood system, it is referred to as ‘clonal haematopoiesis’ (CH) or ‘clonal haematopoiesis of indeterminate potential’ to distinguish these cellular expansions from overt haematological disease [1]. The incidence of detectable CH increases with age, with relatively large clones detected using standard next-generation sequencing occurring in >10% of the population by 70 years of age [2]. However, with the advent of error-corrected DNA sequencing, more recent studies have shown that smaller clones can be detected in most individuals by middle age and that this condition is an inevitable consequence of ageing [3,4].

CH was initially inferred from studies that detected the skewing of X chromosome inactivation in elderly females free from haematological disorders [5]. Subsequent studies revealed that many of these clonal events can be associated with mutations in dozens of driver genes (eg, *DNMT3A, TET2, ASXL1, TP53*, etc.) that are recurrently mutated in haematological malignancies [2,6,7], indicating that the growth of these clones represents a precancerous state. A major advance in the field occurred when it was recognised that individuals with relatively large CH clones exhibited accelerated mortality, predominantly attributed to an increase in cardiovascular disease [2]. Since these early reports, many studies have linked CH with mortality and a wide variety of diseases, including coronary heart disease and various forms of heart failure (Fig 1).

**Figure 1:**
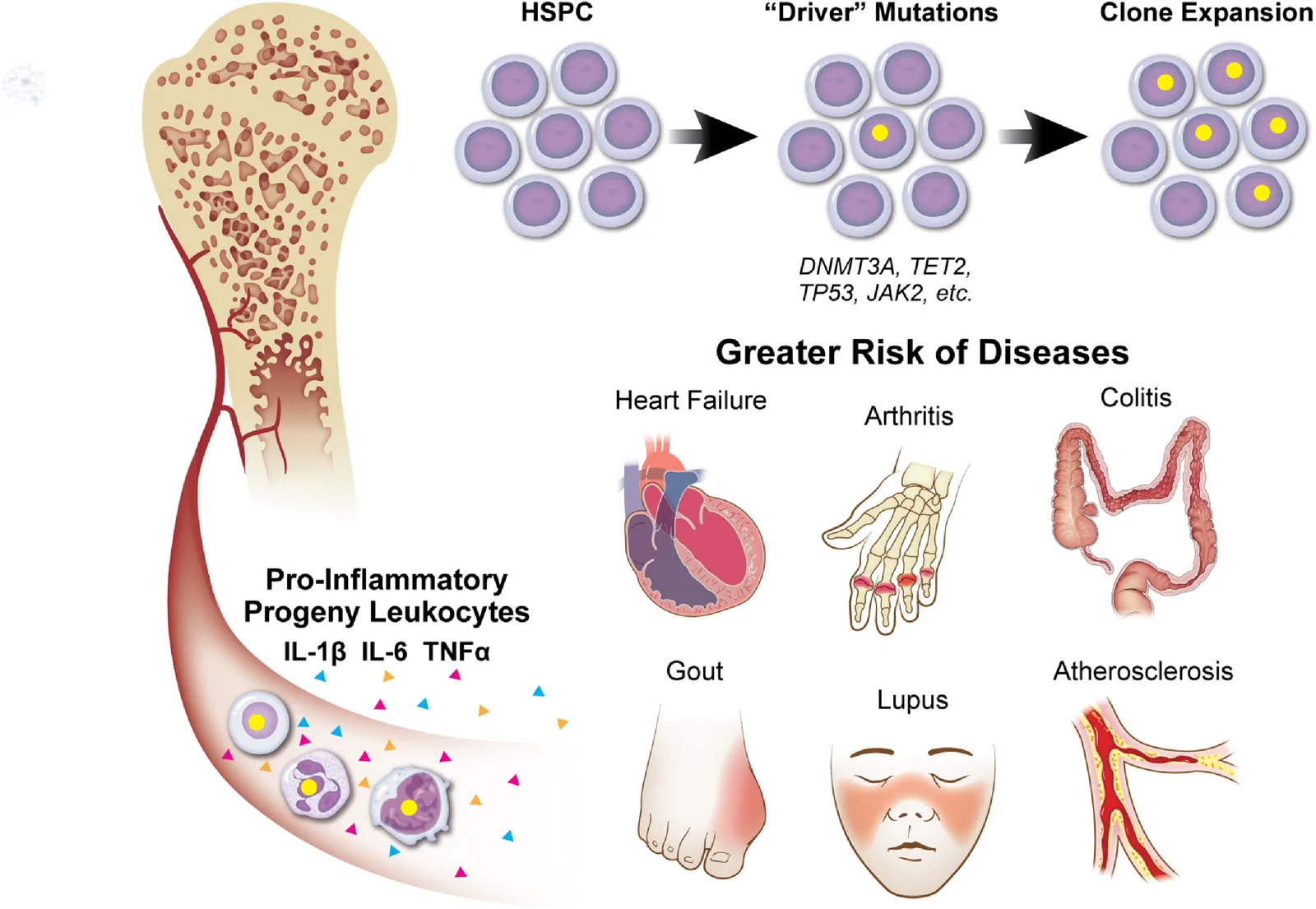
Clonal haematopoiesis promotes systemic inflammation and increases the risk of immune-mediated diseases. Somatic driver gene mutations (eg, *DNMT3A, TET2, TP53*, and *JAK2*) in haematopoietic stem and progenitor cells (HSPCs) lead to clonal expansions in the bone marrow niche. The expanding mutant clones increasingly give rise to progeny leukocytes that harbour the mutation. The mutant leukocytes produce elevated levels of proinflammatory cytokines, including interleukin (IL)-1β, IL-6, and tumour necrosis factor α (TNFα). This chronic inflammatory state may contribute to the risk of developing autoimmune, cardiovascular, and other inflammation-related diseases.

Experimental studies have provided evidence for causal links between CH and disease and have defined aspects of the mechanism. In the study of Fuster et al [8], CH was modelled by performing a competitive bone marrow transplantation with a mutant form of the *Tet2* gene, which is analogous to the inactivating mutations in the *TET2* driver gene that occur in human CH. As expected, these mutant cells could outcompete the wild-type cells, leading to their expansion. However, blood cell counts did not change, which is generally consistent with the clinical paradigm of CH. Notably, this condition led to more atherosclerotic lesions when this CH model was established in hyperlipidemic mice. Additional analyses revealed that leukocytes with the haematopoietic Tet2 mutation displayed a heightened inflammatory state. Specifically, the Tet2 mutation led to activation of the NLRP3 inflammasome, giving rise to greater interleukin (IL)-1β and downstream IL-6 production. Treating mice with an inflammasome inhibitor reversed the accelerated atherosclerosis that was caused by the CH condition. Subsequent experimental studies have shown that this proinflammatory mechanism is shared by multiple CH driver genes, including *DNMT3A, ASXL1, JAK2, TP53*, and *PPM1D*, and the elevated inflammatory state can be observed in multiple disease model systems [1].

Studies have shown that individuals with detectable CH display an elevated inflammatory state. Notably, the CANTOS trial (Canakinumab Antiinflammatory Thrombosis Outcome Study) examined escalating doses of a neutralising IL-1β antibody (canakinumab) in postmyocardial infarction patients with high levels of the inflammation marker C-reactive protein. It was found that targeting IL-1β at a middle dose resulted in a 15% reduction in major adverse cardiovascular events (MACE) compared with the placebo group [9]. In a post hoc analysis, it was found that individuals with CH mutations in TET2 displayed a 62% reduction in MACE in response to canakinumab therapy at any dose, whereas individuals without CH had a 7% reduction in MACE in response to therapy [10]. Thus, assaying for CH in the patient population could serve to identify superior responders to immunosuppressive therapies.

Several studies have detected CH in patients with autoimmune disease. In an analysis of the UK BioBank, CH was associated with higher risks for autoimmune diseases, including incident Crohn’s disease, psoriasis, rheumatoid arthritis (RA), and vasculitis [11]. These CH carriers also exhibited increased levels of inflammatory markers (Fig 1). In a pooled analysis of the National Institutes of Health All of Us Research Programme, Vanderbilt BioVU, and UK BioBank, CH was associated with an increased risk of incident seropositive, but not seronegative, RA [12]. In a related study, CH was enriched in individuals with RA in the FINRISK and FinnGen cohorts, and this study provided evidence for distinct driver gene-dependent associations between CH and distinct RA subtypes [13]. CH is also reported to be enriched in patients with systemic lupus erythematosus [14] and systemic sclerosis [15]. It has also been reported that CH prevalence is significantly elevated in patients with inflammatory bowel disease, particularly Crohn’s disease and ulcerative colitis, where the presence of these mutant clones may correlate with more aggressive disease phenotypes and an altered response to targeted therapies [16]. As various CH driver genes have been linked to inflammatory mechanisms, one can posit that the CH condition contributes to disease severity by elevating systemic inflammation. Alternatively, the elevated inflammatory state caused by the autoimmune disease could be a feature that stresses the haematopoietic stem cell environment, thereby accelerating the expansion of the mutant clones. Further clinical and experimental studies are needed to assess the causality and directionality of CH in autoimmune disorders.

It is becoming apparent that inflammation resulting from CH impacts nearly the entire spectrum of rheumatological and immunological disorders. Although much progress has been made, challenges remain in our understanding of how mutant haematopoietic clones augment disease processes [17]. For example, there is limited information on what constitutes a clinically relevant clone size and how this differs among the different driver gene candidates. Although largely overlooked, it is also recognised that many of the clonal events detected in analyses of blood cannot be attributed to the currently known driver genes [4,6]. Additional clinical and experimental studies in many disease systems will be required to address these outstanding issues.

## CRediT authorship contribution statement

**Kenneth Walsh:** Funding acquisition, Supervision, Writing – review & editing. **Ariel H Polizio:** Writing – review & editing.

## Competing interests

The authors have no competing interests to declare.
